# Mid-Luteal Olfactory Abilities Reveal Healthy Women’s Emotional and Cognitive Functions

**DOI:** 10.3389/fnins.2022.826547

**Published:** 2022-01-31

**Authors:** Fangshu Yao, Kepu Chen, Yiyun Zhuang, Xueer Shen, Xiaochun Wang

**Affiliations:** ^1^School of Psychology, Shanghai University of Sport, Shanghai, China; ^2^State Key Laboratory of Brain and Cognitive Science, CAS Center for Excellence in Brain Science and Intelligence Technology, Institute of Psychology, Chinese Academy of Sciences, Beijing, China

**Keywords:** olfactory function, menstrual cycle, healthy women, emotional symptoms, behavioral impulsivity

## Abstract

Ovarian hormones modulate women’s physical and psychological states periodically. Although the olfactory function is increasingly recognized as a reflection of physical and mental health conditions in the clinic, the role of olfaction in emotional and cognitive functions for healthy individuals has yet to be elucidated, especially when taking the menstrual cycle into account. We carried out a comprehensive investigation to explore whether the menstrual cycle could modulate women’s olfactory function and whether healthy women’s emotional symptoms and behavioral impulsivity could be characterized by their olfactory abilities at a specific menstrual cycle stage. Twenty-nine healthy young women were evaluated repeatedly using a standard olfactory test battery during the late follicular and mid-luteal phases. Their emotional symptoms and behavioral impulsivity were separately quantified via psychometric scales and a stop-signal task. We observed enhanced olfactory discrimination performance during the mid-luteal phase than the late follicular phase. We also found that women’s better olfactory discrimination and worse olfactory threshold in the mid-luteal phase predicted fewer individual emotional symptoms and lower behavioral impulsivity, respectively. These relationships were nonetheless absent in the late follicular phase. Our data extend previous clinical observations of the coexistence of olfactory deficits and neuropsychiatric disorders, providing new insights into the significance of olfaction and ovarian hormones for emotional and cognitive functions.

## Introduction

The amygdala and orbitofrontal cortex are not only olfactory processing regions but also emotion and cognition regulatory structures, thereby potentially drawing connections between olfactory function, emotion regulation, and cognitive control (Heimer et al., 2007; Kohli et al., 2016). Studies on patients with psychiatric (e.g., schizophrenia and depression) and neurodegenerative (e.g., Parkinson’s disease and Alzheimer’s disease) disorders have discovered that hyposmia often accompanies emotional symptoms (Martzke et al., 1997; Croy et al., 2014; Kohli et al., 2016; Croy and Hummel, 2017) and cognitive decline (Masala et al., 2018; Sanna et al., 2021). Although olfactory identification and recognition performances were reported to be comparable between patients with mild or moderate depressive disorder and healthy controls (Zucco and Bollini, 2011), decreased odor discrimination ability was associated with a more impulsive tendency even within the non-clinical population (Herman et al., 2018). Recent studies further reveal the indicative role of olfaction in humans. Individual olfactory perception was demonstrated to be unique and reliable, reminiscent of fingerprints revealing genetic information (Secundo et al., 2015). In addition, human sniffing behavior could promote both olfactory and non-olfactory cognitive task performances, such as olfactory memory consolidation and visuospatial imagination (Arshamian et al., 2018; Perl et al., 2019). Based on the above findings, we intended to shed some light on the role of olfaction in emotional and cognitive functions among healthy individuals.

Ovarian hormones enable women to outperform men in various olfactory tests (Doty and Cameron, 2009). However, these ovarian hormonal levels are not constant but fluctuate with the menstrual cycle, potentially leading to corresponding changes in olfactory perception. There are quite a few results concerning the menstrual cycle variation in olfactory sensitivity (Hummel et al., 1991; Watanabe et al., 2002; Martinec Nováková et al., 2014). It was even reported that epithelial cell turnovers in the nasal cavity and vagina were almost synchronous (Navarrete-Palacios et al., 2003). We intended to examine the role of the menstrual cycle on women’s olfactory function. Odor threshold, discrimination, and identification abilities were repeatedly measured during two representative menstrual phases: the late follicular (high estrogen and low progesterone) and mid-luteal (high estrogen and high progesterone) phases.

Meanwhile, the menstrual cycle was suggested to modulate women’s cognitive function and emotional processing to a certain extent (Sundstrom Poromaa and Gingnell, 2014). It is, therefore, necessary to take the menstrual cycle into account when investigating the relationship between olfactory, emotional, and cognitive functions among female adults. Anxiety and depression symptoms reflect a failure to cope with negative emotions and can be predicted from emotion regulation strategies (Martin and Dahlen, 2005; Hofmann et al., 2012a). Thus, they were measured by the Beck Anxiety Inventory (BAI) and Beck Depression Inventory (BDI-13) separately and were regarded as indicators of the emotional function in the current study. The stop-signal task was adopted to measure one’s behavioral impulsivity or response inhibition (Castiglione et al., 2019; Verbruggen et al., 2019). In this task, relatively longer stop-signal delay (SSD) and shorter stop-signal response time (SSRT) indicate a less impulsive tendency and better response inhibition, subserving successful self-regulation (Hofmann et al., 2012b). Taken together, this study aimed to compare female olfactory functions between different menstrual phases. We also would like to explore the potential role of specific olfactory ability in emotional symptoms and behavioral impulsivity among healthy female individuals, with consideration of the menstrual cycle.

## Materials and Methods

### Participants

We estimated the sample size *a priori* using the G*power 3.1.9.2 (Faul et al., 2007) with a moderate effect size *d* of 0.5, α error at 0.05, and power set at 0.8, yielding a sample size of 34 subjects. Thus, we preliminarily recruited 36 female college students in this study through online advertisement. All participants reported a regular menstrual cycle, no pregnancy or breastfeeding, no history of psychiatric or hormonal disorders, and no administration of oral contraceptives or other hormonal medications during the past 6 months. After recruitment, all participants were assessed with the Beck Anxiety Inventory (BAI), Beck Depression Inventory (BDI-13), and Premenstrual Symptoms Screening Tool (PSST) to quantify their anxiety, depression, and premenstrual syndrome symptoms, respectively. One of them withdrew due to scheduling conflicts. Another four were finally excluded for irregular menstruation that interfered with proper testing time points. Two more participants were excluded due to meaningful clinical depression and anxiety symptoms (both BDI-13 and BAI scores ≥10) (Orff et al., 2007). A total of twenty-nine healthy participants were included for final analyses (mean age ± SD = 20.45 ± 1.70 years; mean BMI ± SD = 19.39 ± 2.34 kg/m^2^; mean cycle length ± SD = 30.10 ± 2.51 days). They showed no clinically significant anxiety (BAI = 3.86 ± 3.45) and depression (BDI-13 = 2.90 ± 2.73) symptoms. Besides, they had only mild or no premenstrual syndrome symptoms, as revealed by the PSST score (11.38 ± 8.28). All participants provided written informed consent before the study and obtained their participation fee when the whole testing procedure was finished. This study was conducted under the Declaration of Helsinki and approved by the Ethics Committee of Shanghai University of Sport.

### Experiment Procedure

**FIGURE 1:**
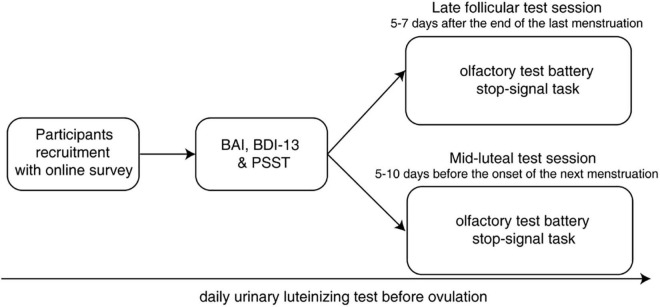
Experimental procedure of the current study. BAI, Beck Anxiety Inventory; BDI-13, Beck Depression Inventory; PSST, Premenstrual Symptoms Screening Tool.

After completing all the psychometric scales, these participants were informed of the next inspection time according to their menstrual history. Test sessions were scheduled during the late follicular (5–7 days after the end of the last menstruation: 5.76 ± 1.08 days) and mid-luteal (5–10 days before the onset of the next menstruation: 7.45 ± 2.52 days, determined based on their menstrual history and urinary luteinizing hormone surge) phases. Participants were repeatedly measured by a standard olfactory test battery and a behavioral explicit emotional stop-signal task in each test session in a counterbalanced manner (Figure 1). As a result, 15 of them performed their first test in the late follicular phase while the remaining 14 participants performed their first test in the mid-luteal phase, with these two sessions separated by at least 1 week (17.03 ± 7.32 days). Before each session, participants were asked to abstain from alcohol and caffeine for 24 h and strictly fast for at least 3 h.

### Questionnaires

#### Beck Anxiety Inventory and Beck Depression Inventory

The BAI is a 21-item self-reported questionnaire developed to assess physical and cognitive symptoms of anxiety (Ulusoy et al., 1998). The score for each item ranges from 0 to 3 on a 4-point scale (total score range: 0–63), with a higher score indicating more severe anxiety symptoms. The BDI-13 is a 13-item short form of the Beck Depression Inventory providing information on the severity of depression (Beck and Beamesderfer, 1974). The total BDI-13 score ranges from 0 to 39, with a higher score indicating more evident depression.

#### Premenstrual Symptoms Screening Tool

The PSST is developed based on DSM-IV criteria to evaluate premenstrual syndrome symptoms (Steiner et al., 2003), with a higher score indicating more severe symptoms. It consists of 19 items, including a list of premenstrual symptoms and a measure of impairment, whose score ranges from 0 to 57.

### Standard Olfactory Test Battery

We adopted a native Chinese olfactory test battery (including olfactory threshold, discrimination, and identification tests) developed by the Institute of Psychology, Chinese Academy of Sciences. The protocol for the odor threshold test was adapted from Ghorbanian et al. (1983), including 20 felt-tip pens filled with a dilution series of eugenol in decreasing order (from 4% v/v to 7.63 × 10^–6^% v/v) and a blank pen filled with pure solvent. Participants were asked to indicate which pen produced a more substantial sensation when presented successively with one odorized pen and one blank pen in random order. The test started from the 12th pen (1.95 × 10^–3^% v/v). The difficulty (i.e., the number of pens) was varied according to a standard seven-reversal staircase procedure (Doty et al., 2001): two successive correct answers triggered a step forward, and one incorrect answer triggered a step backward. A reversal point was defined as any change in the direction of the staircase. Ultimately, the last four staircase reversal points were averaged as the odor threshold score, such that a higher score corresponded to better odor detection ability. The odor discrimination test comprised 16 triplets of odorized pens, with two identical and one different. Participants were asked to indicate the odd one after sampling all three odorants randomly. The number of correct trials was regarded as the odor discrimination score. During olfactory threshold and discrimination tests, participants were blindfolded to prevent any possible visual cues. The odor identification test included 16 different odorized pens selected from the Chinese Smell Identification Test (Feng et al., 2019). Participants were asked to select the optimum description from four options for each odorant. The number of correct trials was regarded as the odor identification score. The duration of each odor presentation was 2 s, delivered at 30-s intertrial intervals to avoid olfactory fatigue.

### Visual Stimuli and the Explicit Emotional Stop-Signal Task

As fluctuating ovarian hormones were suggested to modulate women’s food craving and their brain activation pattern to visual food stimuli (Alonso-Alonso et al., 2011; Klump et al., 2013), we speculated that this modulation effect might involve food-related behavioral impulsivity. Therefore, both positive and negative food images were used as targets in our explicit emotional stop-signal task (Ding et al., 2020). These food images were taken from a cross-culture food image database CROCUFID (Toet et al., 2019). According to the valence rating results (1 = very disgusting, 9 = very delicious) from another cohort of 30 participants (22 females, mean age ± SD = 24.00 ± 3.97 years), we picked out two categories of images that differed significantly in valence (positive = 6.91 ± 0.56; negative = 1.50 ± 0.44). All visual stimuli were presented through the Psychophysics toolbox extension of MATLAB (^®^MathWorks) on a 24-inch LED monitor and were viewed by participants from a distance of 60 cm.

**FIGURE 2:**
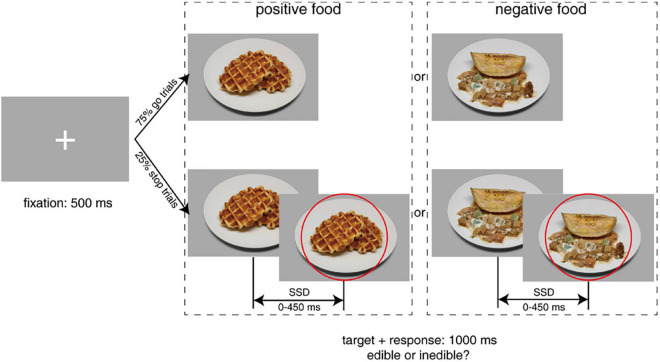
Schematic overview of the explicit emotional stop-signal paradigm. Participants were asked to classify food stimuli as edible or inedible as quickly as possible in go trials. In stop trials, they had to withhold the response when they saw the stop signal (a red circle). Standardized food images were taken from the CROCUFID database available from the OSF repository at https://osf.io/5jtqx. SSD, stop-signal delay.

The explicit emotional stop-signal task paradigm was adapted from a previous study (Ding et al., 2020; Figure 2). Participants were instructed to distinguish between different food categories via a speeded button press after food image presentation (go trials). In a small subset of trials (25%), a stop signal (red circle) appeared after a variable SSD, and they were required to withhold their response at that condition (stop trials). Each trial started with the presentation of a 500-ms white central fixation cross against a light gray background [RGB: (169, 169, 169)]. Following the fixation cross, a food image subtending 20° × 20° appeared for 1,000 ms in the middle of the screen. Participants were asked to classify the presented food as edible or inedible by pressing “F” (with the left hand) or “J” (with the right hand) on the keyboard as quickly as possible. The trial was terminated once a valid response was registered or the food image disappeared, after which a new trial would begin following a 500-ms intertrial interval. Each participant completed a total of 512 trials of this stop-signal task, equally divided into four separate experimental blocks. We adopted a tracking procedure to adjust SSD (initially set to 250 ms and varied between 0 and 450 ms in each block) according to participants’ current performance (Verbruggen et al., 2019). Specifically, SSD decreased after an unsuccessful stop trial and increased after a successful stop trial in a 50-ms step, separately for negative and positive food images. Before data collection, several practice blocks (24 trials per block) with jittered SSD (150–250 ms) and trial-by-trial feedback were introduced to inform participants whether their response was appropriate. Assignment of left and right keys was counterbalanced across participants and menstrual phases to minimize the practice effect.

### Data Analyses

Data distribution was checked by the Shapiro–Wilk normality test. Paired-sample *t*-tests (for normal distributions) and paired-sample Wilcoxon signed-rank tests (for skewed distributions) were used to compare odor threshold, discrimination, identification, and total TDI scores between the late follicular and mid-luteal phases. SSRT was calculated via the integration method (Verbruggen et al., 2013), according to the distribution of go RT. We first replaced omitted go trials with the maximum RT (1,000 ms). We then subtracted the mean SSD from the estimated finishing time of the stop process (i.e., the nth RT in the distribution where n represents the proportion of unsuccessful stop trials multiplied by the number of RTs in the distribution). We used intraclass correlation coefficients (ICCs) to assess the test-retest reliability for correct go RT, incorrect stop RT, SSD, accuracy in stop trials, and SSRT estimation. Mean SSD and estimated SSRT were analyzed as dependent variables in repeated-measures ANOVAs with food valence (negative vs. positive) and the menstrual cycle (late follicular vs. mid-luteal) as within-subject factors. Due to the partly non-normal distribution of the data, we performed the Spearman rank correlation analyses to explore the relationships between emotional symptoms (BAI and BDI-13), behavioral impulsivity (SSD and SSRT), and different olfactory functions (threshold, discrimination, and identification). For the exploratory purpose, *p*-values were not corrected for multiple comparisons in correlation analyses. A *p*-value less than 0.05 was considered statistically significant. These significant variables were subsequently entered into linear regression models to separately predict emotional symptoms and behavioral impulsivity.

## Results

### Olfactory and Stop-Signal Task Performances Between Different Menstrual Phases

In the olfactory test battery, our participants obtained a significantly higher odor discrimination score during the mid-luteal phase than the late follicular phase [luteal vs. follicular: 11.79 ± 1.70 vs. 10.97 ± 1.84, *t*(28) = 2.36, *p* = 0.026, Cohen’s *d* = 0.44]. However, we did not notice any significant difference in odor threshold (luteal vs. follicular: 13.35 ± 4.02 vs. 12.85 ± 4.19, Wilcoxon signed-rank *p* = 0.97), identification (luteal vs. follicular: 14.62 ± 0.90 vs. 14.76 ± 0.87, Wilcoxon signed-rank *p* = 0.31), and total TDI scores [luteal vs. follicular: 39.77 ± 4.37 vs. 38.58 ± 5.22, *t*(28) = 1.22, *p* = 0.23].

Our participants scored 91.9 ± 3.7% of go trials and 62.9 ± 9.3% of stop trials in the stop-signal task. The mean response time (RT) of go trials (636 ± 65 ms) was significantly longer [*t*(28) = 13.44, *p* < 0.001, Cohen’s *d* = 2.50] than that of unsuccessful stop trials (575 ± 46 ms) and was uncorrelated (ρ = −0.02, *p* = 0.91) with SSRT (252 ± 33 ms), suggesting that a valid tracking procedure was performed and the independent race model assumption was not violated (Verbruggen and Logan, 2009). The test-retest reliability of the stop-signal task was acceptable with fair to good ICCs according to Cicchetti’s guidelines (0.73 for correct go RT, 0.60 for incorrect stop RT, 0.73 for SSD, 0.72 for accuracy in stop trials, and 0.46 for SSRT estimation) (Cicchetti, 2001; Congdon et al., 2012). We did not observe any effect of the menstrual phase on mean SSD and SSRT, neither in the main effects nor the interactions (*p* > 0.2). Therefore, they were grand averaged across food valence and menstrual cycle conditions in the following analyses.

### Mid-Luteal Olfactory Discrimination Ability Reveals Healthy Women’s Emotional Symptoms

**FIGURE 3:**
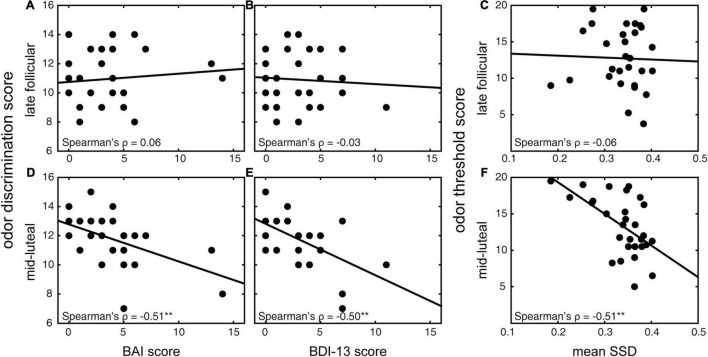
Relationships between olfactory discrimination and emotional symptoms and between olfactory threshold and behavioral impulsivity in different menstrual phases. **(A,D)** Correlations between BAI and odor discrimination scores in the late follicular **(A)** and mid-luteal **(D)** phases. **(B,E)** Correlations between BDI-13 and odor discrimination scores in the late follicular **(B)** and mid-luteal **(E)** phases. **(C,F)** Correlations between SSD (in seconds) in the stop-signal task and odor threshold score in the late follicular **(C)** and mid-luteal **(F)** phases. **p < 0.01 (uncorrected).

We first investigated whether healthy women’s olfactory function at a specific menstrual stage could reveal their emotional symptoms. Exploratory Spearman correlation analyses were first used to quantify the relationship between emotional symptoms and different olfactory functions (Table 1). Both BAI and BDI-13 scores were significantly negatively correlated with the odor discrimination score in the mid-luteal phase (ρ = −0.51 and −0.50, *p* = 0.005 and 0.006, respectively; Figures 3D,E). These relationships were nonetheless absent in the late follicular phase (ρ = 0.06 and −0.03, *p* = 0.77 and 0.90, respectively; Figures 3A,B). We also observed a significant positive correlation between BDI-13 and the odor identification score in the mid-luteal phase (ρ = 0.52, *p* = 0.004) but not in the late follicular phase (ρ = 0.27, *p* = 0.16). Then we conducted linear regression analyses with the luteal odor discrimination score to predict anxiety symptoms and luteal odor discrimination and identification scores to predict depression symptoms (Table 2). The BAI score was significantly predicted by the odor discrimination score in the mid-luteal phase (β = −0.52, *p* = 0.004). In the regression model for the BDI-13 score, only the odor discrimination (β = −0.45, *p* = 0.011) but not the odor identification score (β = 0.31, *p* = 0.074) in the mid-luteal phase was statistically significant.

**TABLE 1:** The bivariate relationships (expressed by Spearman’s ρ) between emotional symptoms, behavioral impulsivity, and olfactory functions.

	Late follicular phase			Mid-luteal phase		
	Threshold	Discrimination	Identification	Threshold	Discrimination	Identification
BAI	0.31	0.06	0.17	0.17	−0.51**	0.32
BDI-13	0.30	−0.03	0.27	−0.02	−0.50**	0.52**
SSD	−0.06	0.12	−0.14	−0.51**	0.11	0.07
SSRT	−0.13	−0.03	−0.10	0.27	−0.00	−0.09

*BAI, Beck Anxiety Inventory; BDI-13, Beck Depression Inventory; SSD, stop-signal delay; SSRT, stop-signal response time.*

**p < 0.01 (uncorrected).

**TABLE 2:** Linear regression models predicting emotional symptoms and behavioral impulsivity.

Dependent variable	Predictor	*B*	Standard error	β	*P*
BAI	Mid-luteal discrimination	−1.05	0.34	−0.52	0.004
			*R*^2^ (Adjusted *R*^2^): 0.27 (0.24)		
			*F* (*p*-value): 9.85 (0.004)		
BDI-13	Mid-luteal discrimination	−0.73	0.26	−0.45	0.011
	Mid-luteal identification	0.92	0.50	0.31	0.074
			*R*^2^ (Adjusted *R*^2^): 0.40 (0.36)		
			*F* (*p*-value): 8.71 (0.001)		
SSD	Mid-luteal threshold	−0.007	0.002	−0.57	0.001
			*R*^2^ (Adjusted *R*^2^): 0.32 (0.30)		
			*F* (*p*-value): 12.91 (0.001)		

### Mid-Luteal Olfactory Threshold Ability Reveals Healthy Women’s Behavioral Impulsivity

We then explored whether healthy women’s olfaction function at a specific menstrual stage could reveal their behavioral impulsivity. Before we conducted the regression analyses, we calculated bivariate Spearman correlations for indexes of behavioral impulsivity and different olfactory functions (Table 1). We found that SSD was significantly negatively correlated with odor threshold score in the mid-luteal phase (ρ = −0.51, *p* = 0.005; Figure 3F) but not in the late follicular phase (ρ = −0.06, *p* = 0.77; Figure 3C). In the linear regression model, the mean SSD was significantly predicted by odor threshold score in the mid-luteal phase (β = −0.57, *p* = 0.001, Table 2). At the same time, we found no significant correlation for SSRT (*p* > 0.1) thus we did not conduct a regression analysis.

## Discussion

We investigated whether the menstrual cycle could modulate female olfactory function and whether olfaction was indicative of emotional and cognitive functions among healthy female adults in a stage-specific manner, particularly for emotional symptoms and behavioral impulsivity. Our results demonstrated improved olfactory discrimination ability in the mid-luteal phase and the informative role of olfactory discrimination and threshold abilities at the same stage. More precisely, women’s emotional symptoms and behavioral impulsivity could be predicted by their olfactory discrimination and threshold abilities in the mid-luteal phase, respectively. These findings expand our knowledge about the significance of olfaction in emotional and cognitive functions among the non-clinical population. Moreover, we suggest the mid-luteal phase as a potentially informative stage for early screening for neuropsychiatric-related disorders with sensitive olfactory ability assessment.

### Menstrual Cycle Modulates Female Olfactory Function

Although the menstrual cycle variation in olfactory function has been investigated for decades (Doty et al., 1981; Hummel et al., 1991), most studies were confined to odor sensitivity, and no definite conclusions have been reached yet (Doty and Cameron, 2009; Martinec Nováková et al., 2014). Our result, along with other evidence (Derntl et al., 2013; McNeil et al., 2013), compensates for this deficiency and demonstrates relatively better olfactory discrimination ability in the mid-luteal phase. It also complies with previous findings that female olfactory ability enhanced after oral contraceptive treatment (Derntl et al., 2013) and declined during the withdrawal period (Endevelt-Shapira et al., 2020), suggesting a promoting effect of ovarian hormones in olfaction, especially for high-order olfactory functions like discrimination (Hummel et al., 1997). However, the impact of hormonal contraceptives on the olfactory threshold, a more peripheral olfactory function, seems to vary with odor specificity (Lundstrom et al., 2006; Schaefer et al., 2021). Likewise, the odor threshold result in the current study did not exhibit a phase-dependent change, which may be specific to the odor stimulus we used here, namely eugenol without any biological relevance to humans (Martinec Nováková et al., 2014). Although no difference was found for the odor identification likely due to a practice and a ceiling effect, different modulations of the menstrual cycle on olfactory threshold and discrimination abilities may be attributable to their divergent recruitments of high-level cognitive functions.

### Mid-Luteal Olfactory Abilities Predict Emotional and Cognitive Functions

In our study, mid-luteal olfactory discrimination ability revealed women’s emotional function, indicating that inferior olfactory discrimination performance was associated with more anxiety and depression symptoms. This phenomenon agreed with previous results from patients with major depressive and anxiety disorders (Atanasova et al., 2008; Clepce et al., 2012). In contrast to the evidence of no olfactory dysfunction among patients with mild or moderate depressive disorder (Zucco and Bollini, 2011), our results demonstrated an indicative role of olfactory discrimination ability even among those healthy individuals. However, it is possible that this indicative olfactory function only exhibits at a specific menstrual cycle stage, such as the mid-luteal phase we found here. Although the luteal odor identification score was significantly correlated with the BDI-13 score, it had a limited effect on predicting women’s depression symptoms when entered in the regression model. This erratic relationship was possibly due to a practice effect, as odor identification requires long-term memory to recognize or name the odor (Hummel et al., 1997), and a ceiling effect, as odor identification is relatively easy for healthy young adults.

Meanwhile, mid-luteal olfactory threshold ability was negatively correlated with the stop-signal task performance, indicating that superior olfactory threshold performance was associated with a more impulsive tendency, as reflected by a relatively shorter SSD. Albeit counterintuitive at first sight, this finding was in line with previous studies on attention deficit hyperactivity disorder (ADHD) patients. Those ADHD patients exhibited a supernormal olfactory sensitivity and impaired response inhibition (Romanos et al., 2008; Wolfers et al., 2016), both of which returned to normal after dopaminergic medication (DeVito et al., 2009; Fuermaier et al., 2018). Additionally, low alcohol intake and 24-h fasting have been suggested to improve human olfactory ability, particularly for the olfactory threshold (Cameron et al., 2012; Endevelt-Shapira et al., 2014). Since both alcohol intake and fasting can induce behavioral impulsivity through the weakened prefrontal cortex (Abernathy et al., 2010; Friedman and Robbins, 2021), which also receives direct inputs from the primary olfactory regions, these findings may jointly signify an inhibitory impact from the prefrontal cortex on the original olfactory function.

### Neural Mechanisms Underlying the Informative Mid-Luteal Olfactory Abilities

Based on the above results, mid-luteal olfactory abilities revealed healthy women’s emotional and cognitive functions, which were relatively constant through the menstrual cycle but differed among healthy individuals. Olfactory information derived from the olfactory bulb was first processed by the primary olfactory structures such as the amygdala for odor detection and then further processed by the secondary olfactory cortex such as the orbitofrontal cortex for odor discrimination and identification (Gottfried and Zald, 2005). Both amygdala and orbitofrontal cortex are suggested to be involved in emotion regulation and response inhibition (Banks et al., 2007; Coccaro et al., 2007). Therefore, the shared neural network may underly the relationship between olfactory capacity, emotional and cognitive functions, not only for clinical patients but also for healthy individuals, as the phenomenon we observed here. During the mid-luteal phase, estrogen and progesterone reach a peak level (Draper et al., 2018), with their receptors highly expressed in the brain (Gruber et al., 2002; Brinton et al., 2008). Especially progesterone, whose metabolites interact with the gamma-aminobutyric acid (GABA_A_) receptor to modify cognitive functions (Melcangi et al., 2011). In addition, a decrease in multiple amino acids and derivatives and lipid species was observed in the luteal phase, implying increased anabolic demands during this period (Draper et al., 2018). Accordingly, the luteal phase may serve as an important stage that amplifies differential individual responses to external stimuli, including the evolutionarily ancient olfactory function.

### Limitations and Future Directions

A recent study on a healthy population reported that better olfactory discrimination and worse olfactory threshold abilities predicted a shorter SSRT in a standard stop-signal task (Herman et al., 2018). However, we did not find a significant correlation between olfactory abilities and the SSRT estimation in the current study. The possible reason might be that although the more reliable integration method (Verbruggen et al., 2019) was introduced to avoid overestimation of SSRT, the SSD range (0–450 ms) was still too short for participants to produce a prepotent response to go stimuli (i.e., identify whether the presented food was edible or inedible). Previous work using simulations demonstrated that the SSRT estimation was most reliable when the accuracy in stop trials was close to 50% (Band et al., 2003; Congdon et al., 2012). As reflected by relatively higher accuracy than 50% in stop trials, the SSRT estimation was less reliable than usual. Nevertheless, as a direct measure, the mean SSD is an excellent complement to SSRT (Herrera et al., 2014). Given that our participants were homogenous healthy individuals, relatively longer SSD was more likely to denote a response bias favoring accuracy over speed in the speed-accuracy trade-off, reflecting a more inhibitory and less impulsive response tendency. Future studies should employ a standard stop-signal task or further modulate the SSD range to obtain a more accurate SSRT estimation. Apart from that, our study was based on a relatively small sample, and all our participants were young college students. This small sample size potentially limits our findings to be extended to a broader cohort, such as those depressed patients or parturients. Given a higher prevalence of stress-related psychiatric disorders in women, future clinical studies on female patients should consider the menstrual cycle variation and olfactory ability assessment in more detail. Moreover, we did not include the measurement of ovarian hormone concentrations, which prevented us from exploring the relationship between olfactory abilities and hormonal levels.

## Conclusion

We examined the role of the menstrual cycle on women’s olfactory function in the current study. As a further exploration, we investigated whether healthy women’s olfactory function at a specific stage could reveal their emotional and cognitive functions. Olfactory abilities of threshold, discrimination, and identification were repeatedly measured during two representative menstrual phases: the late follicular and mid-luteal phases. We also measured their emotional symptoms and behavioral impulsivity through psychometric scales and a stop-signal task, respectively. Participants exhibited better olfactory discrimination ability in the mid-luteal phase relative to the late follicular phase. In contrast, no difference between menstrual phases was observed for olfactory threshold and identification abilities. We found that healthy women’s better olfactory discrimination in the mid-luteal phase was associated with fewer emotional symptoms. Their better olfactory threshold performance in the mid-luteal phase was related to increased behavioral impulsivity. The above relationships were nonetheless absent in the late follicular phase. What we have observed extends previous findings of the coexistence of olfactory deficits, emotional symptoms, and cognitive decline in the clinic. Our findings also suggest the olfactory function evaluation in the mid-luteal phase as a potential method to reveal healthy women’s emotional and cognitive functions, which is beneficial for early diagnosis.

## Data Availability Statement

The raw data supporting the conclusions of this article will be made available by the authors, without undue reservation.

## Ethics Statement

The studies involving human participants were reviewed and approved by the Ethics Committee of Shanghai University of Sport. The patients/participants provided their written informed consent to participate in this study.

## Author Contributions

FY, KC, and XW designed the study. FY, YZ, and XS performed testing and data collection. FY analyzed the data and interpreted the results under the supervision of XW. All authors wrote the manuscript and ensured input on the manuscript before approving.

## Conflict of Interest

The authors declare that the research was conducted in the absence of any commercial or financial relationships that could be construed as a potential conflict of interest.

## Publisher’s Note

All claims expressed in this article are solely those of the authors and do not necessarily represent those of their affiliated organizations, or those of the publisher, the editors and the reviewers. Any product that may be evaluated in this article, or claim that may be made by its manufacturer, is not guaranteed or endorsed by the publisher.
